# Naso-alveolar molding for newborn cleft lip and palate

**DOI:** 10.6026/97320630018492

**Published:** 2022-05-31

**Authors:** Rohit Breh, Pankaj Singodia, Shitun Sarangi

**Affiliations:** 1 Department of Dentistry, Tata Main Hospital, Jamshedpur, Jharkhand, India; 2 Department of Plastic Surgery, Tata Main Hospital, Jamshedpur, Jharkhand, India

**Keywords:** Naso-alveolar molding (NAM), pre-surgical infant ortho-pedics, cleft lip, cleft palate, nasal stent

## Abstract

The nasoalveolarmolding (NAM) technique is a relatively new approach to pre-surgical infant orthopedics that reduces the severity of the initial cleft alveolar and nasal deformity before surgery. This technique has proved to be an effective adjunctive therapy for reducing cleft deformity before surgery. Data on NAM are inconsistent with changes in nasal symmetry. However, there is a trend towards a positive effect. Therefore, it is of interest to report clinicians an overview of NAM appliance and method for nasal symmetry assessment to facilitate greater usage of this technique in contemporary practice. Thus, the biomechanics of pre-surgical infant orthopedics and of nasal stent that skillfully shape alveolus and nasal cartilage is explained.

## Background:

Oro-facial clefts can be broadly classified into syndromic and non-syndromic variants. These children require a multidisciplinary care from birth until adulthood and have higher morbidity and mortality [1-2]. Increased frequency of structural brain abnormalities [3-5] and psychological effects on children and families is well proven [6-7]. Standards of care in both developing and developed countries for patients with cleft lip, cleft lip and palate, or cleft palate alone remain a cause for concern [8]. Therefore, it is of interest to report clinicians an overview of NAM appliance and method for nasal symmetry assessment to facilitate greater usage of this technique in contemporary practice.

## Case report:

A newborn male child in the Hospital was referred to the department of dentistry for obturator. On examination, child was medically healthy at birth. Head and neck MRI images reported no significantly abnormal findings. Hearing was not impaired as reported with examinations. Child had a unilateral cleft palate and lip. So, Pre-surgical Naso-alveolar Molding (PSM) was planned before plastic surgeon operates over lip before six months of age. No history of medication, active or passive smoking (or any other form of tobacco habit), no history of trauma or any other significant noticeable pre-natal events.

Head form: Mesocephalic

Face form: Mesoprosopic

Facial symmetry: Asymmetrical

Facial profile: Convex

Lip: Cleft of 12mm unilaterally with left sided upper lip is observed. Right segment of upper lip is positioned sagittally anteriorly as compared to the left lip segment. No abnormality detected with lower lip.

Nose: Nasal tip deviated to the left, short columella length, wide inter-alar width.

Clinical Examination: Intra oral examination: Unilateral cleft lip and palate. Veau's classification group III or Elnassry classification (2007) Class IV are used [5]. No clinical abnormality detected with uvula.

Soft tissue: Frenum attachment to the major segment of the upper lip opposite to the philtrum is studied.

Alveolar segments:Divided into two segments, right larger segment and left shorter segment. Right segment is positioned sagittally anteriorly as compared to left shorter segment.

Teeth: No natal teeth present.

Summarizing the clinical examination: A newborn patient with complete (left) unilateral cleft lip and cleft palate is observed. Cleft at labial segment measuring 12 mm and at alveolar region measuring 11 mm is observed. The cleft lip and alveolar segment over the right side is anterior to the left segment in the sagittal dimension. Nasal tip is deviated to the left with small columella length.

## Objectives of treatment:

[1] Achieving approximation of lip segments.

[2] Achieving approximation of alveolar segments in transverse dimension and in sagittal dimension while maintain the vertical height and distal transverse width of alveolar segments.

[3] Increasing the columella length.

[4] Achieving midline for nasal tip.

[5] Achieving equal alar width and height.

[6] All the objectives to be achieved in less than 6 months of age before the lip repair surgery.

## Treatment plan:

[1] The naso-alveolar molding appliance (NAM) consisting of an intraoral molding plate with nasal stents to mold the alveolar ridge and nasal cartilage concurrently [9-10].

[2] Weekly appointments for adjustments in the molding plates by selectively removing the hard acrylic and adding the soft denture base material to the molding plate.

[3] Incorporating nasal stent when the alveolar gap reduces to 5mm or less. Weekly adjustments in nasal stent to be done with the plate till desired results are achieved.

## Treatment:

Treatment commenced on January 16, 2019 with alveolar molding plate appliance. Date and Stage

[1] January 15, 2019: Maxillary arch impression made with heavy body. Prior to that risk assessment was done by pediatrician and it was decided to take moderate risk consent before taking impression. Impression was taken in Pediatric ICU under all precautions with endoscopic and intubation equipment at alert. Child was positioned head down and impression was made with putty material [11]. January 16, 2019: Alveolar molding plate was prepared with acrylic and delivered. Parents were trained and given instructions for using and maintaining appliance.

[2] January 23 to February 27, 2019: Selective grinding and addition of soft denture liner were added for alveolar molding on weekly basis [12]. Alveolar gap reduced to approx. 6mm, hence nasal stent was planned and a wax model was planned over the plate for construction. March 3, 2019: Nasal stent was made and added to the plate with acrylic [13]. The nasal end was bean shaped covered with a soft liner. It was activated to the minimum when blanch appears at pressure site [14].

[3] March 10 to April 2019: Alveolar molding was continued, and nasal stent was sequentially activated till desired results were achieved. After this parent was instructed continue with the appliance and to visit biweekly for follow up (Figure 1 - see PDF).

[4] June 14, 2019: Lip repair surgery was done by plastic surgeon by Randall-Tennison technique.

[5] January 10, 2020: Palate repair was done.

## Post-treatment Assessment:

Lip approximation and alveolar approximation were accomplished in nearly 14 weeks of commencement of surgery. Lip surgery was done in approximately six months of birth. Inflammation reduced in two weeks period and scar redness started reducing by two months period. Post lip surgery alar base width was symmetrical with adequate columella length and midline symmetry. By one year of age when palate was operated the scar was further reduced and was negligibly visible. One-year follow was done for the patient. Nasal tip was in midline, alar width was maintained, and columella height was adequate. Lip was operated with Randall-Tennison technique and vermillion border alignment was achieved with near invisible scar formation in upper lip [15-16].

## Critical appraisal:

Satisfactory results were achieved considering the facial aesthetics. It is recommended to follow up the case on regular basis, especially when teeth start to erupt. Growth needs to be followed up as scar formation may restrict the growth of maxilla in all the three dimensions [17-21].

## Conclusion:

The NAM technique has shown to improve the surgical outcome of cleft lip and palate compared with other techniques of presurgical orthopedics. Nonsurgical columellar elongation with nasoalveolar molding restored columellar length to near normal and significantly reduced the need for secondary nasal surgery. NAM technique has proved to be an effective adjunctive therapy for reducing hard and soft tissue cleft deformity before surgery. Thus, clinicians are encouraged to undertake further studies with long term follow-ups and randomized control trials in the field of pre-surgical naso-alveolar molding to substantiate the described technique.
